# Potential therapeutic effects of Ebixa, *Ginkgo biloba*, and selenium in a cadmium chloride-induced Alzheimer’s disease manifestations in rats

**DOI:** 10.3389/fnins.2025.1634601

**Published:** 2025-07-23

**Authors:** Afaf Alrikabi, Wasayf Allahyani, Amjad Shaghath, Jawaher Alrashdi, Reem Almoqhem, Fawaz Alasmari, Walid Al-Qerem, Gadah Albasher

**Affiliations:** ^1^Department of Zoology, College of Science, King Saud University, Riyadh, Saudi Arabia; ^2^Department of Biology, College of Science, University of Hail, Hail, Saudi Arabia; ^3^Department of Food Science and Nutrition, College of Food and Agriculture Sciences, King Saud University, Riyadh, Saudi Arabia; ^4^Department of Pharmacology and Toxicology, College of Pharmacy, King Saud University, Riyadh, Saudi Arabia; ^5^King Salman Center for Disability Research, Riyadh, Saudi Arabia; ^6^Faculty of Pharmacy, Al-Zaytoonah University of Jordan, Amman, Jordan

**Keywords:** Alzheimer’s disease, cadmium exposure, cholinergic system, Ebixa, *Ginkgo biloba*, selenium

## Abstract

Alzheimer’s disease (AD) is a devastating neurodegenerative disorder characterized by cognitive decline and neuronal damage. Cadmium exposure has been implicated in AD pathogenesis. This study aimed to investigate the potential therapeutic effects of Ebixa (memantine), *Ginkgo biloba*, and selenium in a cadmium-induced rat model of AD. Adult male Wistar rats were divided into six groups: control, control + Ginkgo-treated, cadmium chloride (CdCl2), CdCl2 + Ebixa-treated, CdCl2 + Ginkgo, and CdCl2 + Ginkgo + Selenium. Behavioral tests, including the Morris water maze and passive avoidance learning, were conducted. Additionally, biochemical analysis of acetylcholine (Ach), choline acetyltransferase (AchT), and acetylcholinesterase (AChE) levels in brain homogenates was performed. Histological sections of the cerebral cortex, cerebellum, and medulla were examined. Apoptotic assessment was conducted using the TUNEL assay. CdCl2 exposure resulted in cognitive deficits, reduced Ach levels, and neuronal damage, mirroring AD-like characteristics. Ebixa treatment improved spatial memory behavior as well as Ach, AchT and AChE levels in the brain. *Ginkgo biloba* and selenium co-administration increased the number of crossings in the Morris water maze test, suggesting memory preservation. Additionally, *Ginkgo biloba* exhibited potential cholinergic system protective effects. Histological analysis revealed neuroprotection in the cerebral cortex, cerebellum, and medulla. TUNEL assays demonstrated anti-apoptotic effects of both Ebixa and the combination of Ginkgo and selenium. Ebixa, *Ginkgo biloba*, and selenium showed promise in mitigating cognitive deficits and preserving neuronal structures in a CdCl2-induced AD manifestation in rats. These findings provide insights into potential therapeutic strategies for AD and warrant further investigation.

## 1 Introduction

Alzheimer’s disease (AD) is a progressive and debilitating neurodegenerative disorder that predominantly affects the elderly. It results in a gradual decline in cognitive, behavioral, and social functioning, significantly impacting individuals’ ability to live independently (Rashid et al., 2020). The primary mechanisms underlying AD include the formation of amyloid plaques and neurofibrillary tangles, leading to neuronal damage, especially affecting cholinergic neurons. Neuroinflammation and oxidative stress are considered potential triggers for AD (Ibrahim et al., 2021). AD is a slow progressive neurodegenerative disorder with a high prevalence among the elderly and has a huge personal and societal impact (Tahami Monfared et al., 2022). The global prevalence of dementia, with AD as a leading cause, is expected to double every 20 years, resulting in a substantial societal and economic burden (Prince et al., 2013).

Currently, there is no curative treatment for AD, and existing allopathic medications are associated with high costs and side effects. As a result, there is a growing need for safe and effective alternative therapies (Thawabteh et al., 2025). Phytoconstituents, such as those found in *Ginkgo biloba* extract, have garnered attention for their potential neuroprotective, antioxidant, and anti-inflammatory properties (Erbil et al., 2008; Zhang et al., 2022; Chen et al., 2024). *Ginkgo biloba* has a long history of medicinal use, and standardized extracts from its leaves are commonly used (Chan et al., 2007; Das et al., 2022; Akaberi et al., 2023). Various medicinal plants, including *Ginkgo biloba*, are being explored for their therapeutic potential against (Nguyen et al., 2025). *Ginkgo biloba* extract, notably EGb761, has been widely studied and is recognized for its potential neuroprotective effects (Zang et al., 2023). In addition, it is believed that *Ginkgo biloba* extract is a potential extract in modulating gut and reversing the impairments of microbial metabolism in mice model of AD (Yu et al., 2023). This extract is one of the most popular herbal supplements and is known for its potential antiapoptotic properties providing neuroprotective effects (Di Meo et al., 2020; Yin et al., 2024).

Additionally, selenium has been studied for its potential role in reducing Alzheimer’s pathology and protecting against neurodegenerative diseases mortality (Du et al., 2016; Tu et al., 2024). It was found that neuroinflammation and neurotoxicity effects were attenuated with resveratrol-selenium treatments in rat models of AD (Abozaid et al., 2022). It was believed that selenium exhibits neuroprotective effects through modulating inflammatory and oxidative stress markers in the brain, particularly in the hippocampus (Liang et al., 2023). The nanoparticles form of selenium had anti-neuroinflammation effects via modulating gut microbiota-NLRP3 inflammasome-brain axis in mice model of AD (Yang et al., 2022). It was suggested that selenium intake could be used as a complementary therapy in patients with migraine since it had positive effects against oxidative stress and associated symptoms in human (Balali et al., 2024). Moreover, selenium could block reactive oxygen species-increased apoptosis using in brain-derived neural progenitor cells (Yeo and Kang, 2007). In addition, selenium inhibits hydrogen peroxide-mediated apoptosis and oxidative stress in the brain of traumatic brain injury animal model (Yeo and Kang, 2007).

Given the significant challenges in treating AD and the potential of these natural compounds, this study aims to investigate the interactive effect of *Ginkgo biloba* extract and selenium on neurobehavioral changes in male rats associated with clinical manifestations of AD. This research study on the interactive effect of *Ginkgo biloba* extract and selenium in mitigating neurobehavioral and molecular changes induced by CdCl2 in male rats. It is hypothesized that the combined treatment of *Ginkgo biloba* extract and selenium will demonstrate a synergistic effect in ameliorating neurobehavioral changes associated with AD in male rats compared to animals treated with only either one of them. Our work also provides information abourt the effects of *Ginkgo biloba* extract and selenium on cholinergic system in the brain. This study examines the impact of *Ginkgo biloba* extract, selenium and the combined treatments on the histology of the brain regions in male rats developed neurobehavioral changes and cholinergic alterations observed in AD models. Overall, the current study aims to provide valuable insights into the potential therapeutic benefits of *Ginkgo biloba* extract, selenium and their combinations in attenuating the progression of AD in pre-clinical phase.

## 2 Materials and methods

### 2.1 Experimental animals

Thirty-six adult male Wister rats (200 ± 20 g, 10 weeks old) were obtained from the College of Sciences, King Saud University (KSU), Riyadh, Saudi Arabia. The rats were housed in controlled conditions (22 ± 2°C, 50% humidity, 12/12 h light/dark), with free access to their designated diets and drinking water. The experimental protocol was approved by the institutional review board of King Saud University (Ref. No.: KSU-SE-21-69).

### 2.2 Experimental design and treatments

The rats were divided into 6 groups (*n* = 6/group):

1. Control Group: Administered an equivalent volume of 0.1 DMSO as a vehicle for 4 weeks.
2. *Ginkgo Biloba*-treated Group: Rats were administered with *Ginkgo Biloba* (100 mg/kg/day, PO) (Winter, 1991) for 4 weeks.
3. CdCl2-treated Group: Rats were given CdCl2 (1.5 mg/kg/day, i.p) for 4 weeks (Hao et al., 2020) (PMID: 32739455).
4. CdCl2 + Ebixa-treated Group: Rats were treated with CdCl2 (1.5 mg/kg/day, i.p) + Ebixa (30 mg/kg/day, i.p) (Li et al., 2013) for 4 weeks.
5. CdCl2 + *Ginkgo Biloba* Group: Rats were administered with CdCl2 (1.5 mg/kg/day, i.p) + *Ginkgo biloba* (100 mg/kg/day, PO) for 4 weeks.
6. CdCl2 + *Ginkgo Biloba* + Selenium Group: Rats were administered with CdCl2 (1.5 mg/kg/day) + *Ginkgo biloba* (100 mg/kg/day, PO) + Selenium (0.5 mg/kg/day, PO) (Ghaffari et al., 2011) for 4 weeks.

### 2.3 Behavioral tests

#### 2.3.1 Morris water maze (MWM)

The assessment of spatial memory was performed using the MWM during the last week of the experiment. The MWM test measures the ability of rats to remember a hidden fixed-location escape platform. Briefly, this test assesses the rats’ memory for a hidden fixed-location escape platform in a swimming pool (diameter = 1.6 m; depth = 60 cm) that is located 2 cm below the water’s surface. The test consists of five training days and a hidden platform. The pool is separated into four quadrants: west, south, east, and north. The hidden platform was placed in the southern-west quadrant to ensure that the swimming distance is equal. The rat was released from one quadrant to locate the hidden platform during three trials (each lasting 90 s) per day. Escape latency (time to find the platform) was recorded as a marker of memory function. Furthermore, on day six, an additional probe trial was carried out in which the hidden platform was taken away, and the number of times the rats crossed the platform’s location was noted (i.e., the rats’ capacity to recall the location of the rescue platform) (Vorhees and Williams, 2006).

#### 2.3.2 Passive learning avoidance (PAL) tests

Short and long-term memory function was tested using the PAL test, which involved exposing rats to an electrical foot shock and measuring their avoidance of the dark room as described previously (Nassiri-Asl et al., 2012). A wooden apparatus (50 × 50 × 35 cm) with two rooms—one large and lit and the other tiny and dark—separated by a door was employed for the test. An electrical stimulator was linked to the floor of the tiny, dimly lit room. There are two stages to the test: the investigation phase and the testing phase. The rats were put in a big room with a door open throughout the exploration phase, which consisted of five trails lasting 5 min each, separated by 30 min. They were then free to enter and exit the darkroom. In the final experiment, the rats were given an electrical foot shock (50 Hz, 1.5 mA/ 1 s) after the door was closed and they entered the dark room. After that, every rat was put back in its cage, and the testing process (testing with an electrical shock) was repeated after 2 h of the last electrical shock trail. As a result, it was noted how long it took each animal to enter the darkroom.

### 2.4 Brains collection and processing

#### 2.4.1 Brains harvesting

After the PAL test, rats were anesthetized using a combination of and 20 mg/mL xylazine and 50 mg/mL ketamine followed by cervical dislocation, and their brains were collected. Six brains per group were fixed in 10% buffered formalin for histological staining, while other brains were snap-frozen for further studies. The hippocampus of all other brains were quickly dissected by an expert pathologist, snap-frozen in liquid nitrogen, and used later for biochemical and molecular studies.

#### 2.4.2 Apoptosis determination

TUNEL Assay was performed in the cerebral cortex of all groups to recognize apoptotic nuclei in brain sections according to the manufacturer’s instructions.

#### 2.4.3 Preparation of brain homogenates

Frozen hippocampus samples were homogenized in phosphate-buffered saline and centrifuged, and the supernatants were collected for biochemical analysis. Protein levels in the brain homogenates were determined using a Bio-Rad substrate reagent and standard proteins.

#### 2.4.4 Determination of Ach levels in the hippocampus

Acetylcholine (Ach) levels were determined using an ELISA kit as explained previously (Huang et al., 2019). Briefly, 50 μL of the standard, blank (0.0 mg/mL standard), other standards, and samples were added to the designated wells in the pre-coated 96-well plate. Next, 50 μL of detection reagent A was added to each well and incubated for 1 h at 37°C. The solutions were aspirated from all wells, and each well was rinsed three times with 350 μL of 1X washing solution using a multi-channel pipette. All wells were entirely emptied of fluids. Subsequently, 100 μL of the detection reagent B working solution was added to each well and incubated for 30 min at 37°C while covering it with the Plate sealer. The plate was rinsed three times with the washing buffer. Subsequently, 90 μL of the substrate solution was added to all wells and incubated for 20 min at 37°C in the dark. Ultimately, 50 μL of the stopping solution was introduced to every well and the absorbance was measured at 450 nm. The level of Ach was indicated as pg./g protein.

#### 2.4.5 Determination of ach, AchT and AChE levels in the hippocampus

Choline transferase (AchT) and acetylcholinesterase (AChE) activities were determined by an ELISA kit for rats according to the manufacture instructions (Gearhart et al., 2006; Huang et al., 2019). For AchT assay, 100 μL of the samples or standards were added to the designated wells of the pre-coated 96 well plate. The dish was sealed and incubated for 1 h at 37°C. The fluids were subsequently removed from all wells, and 100 μL of detection reagent A was added to each well, followed by incubation for 1 h at 37°C. The liquids from each well were removed, and all wells underwent three wash cycles with 350 μL of 1X washing solution using a multi-channel pipette. The plate was dried by tapping it onto an absorbent paper and pouring off the excess. Subsequently, 100 μL of the working detection reagent B was added to every well and allowed to incubate for 30 min at 37°C. The plate was subsequently rinsed three times with the washing buffer. Subsequently, 90 μL of the substrate solution was added to every well and incubated for 20 min at 37°C in the dark. Finally, 50 μL of the stop solution was added to each well, and the absorbance (ABS) was measured immediately at 450 nm. For the AChE experiment, 100 μL of standards or samples were added to their corresponding wells in the pre-coated 96-wells ELISA plate. 100 μL of PBS (pH 7.0–7.2) was used as the blank. 10 μL of the balance solution was applied just to the sample wells. All of the wells, with the exception of the blank well, received 50 μL of the given conjugate, which was then mixed by pipetting. The dish was kept at 37°C for an hour. After that, the wells were rinsed three times using 350 μL of washing buffer. The plate was dried by pounding it on absorbent paper. In a dark environment, 50 μL of substrate A and 50 μL of substrate B were added to each well, and the mixture was incubated for 20 min at 37°C. Following that, the absorbance was measured at 450 nm after adding 50 μL of the stop solution to each well. The levels of AchE were expressed as pg./g, and the concentrations of AchT was expressed as U/g protein.

### 2.5 Statistical analysis

Statistical analyses were conducted using Graph Pad Prism (version 6) with one-way ANOVA followed by Tukey’s *post hoc* test, and similar analysis was performed between groups within each day in escape latency measurements. Significance was set at *p* < 0.05, and data were presented as means ± standard deviation (SD).

## 3 Results

### 3.1 The behavior’s test

**Figure 1:**
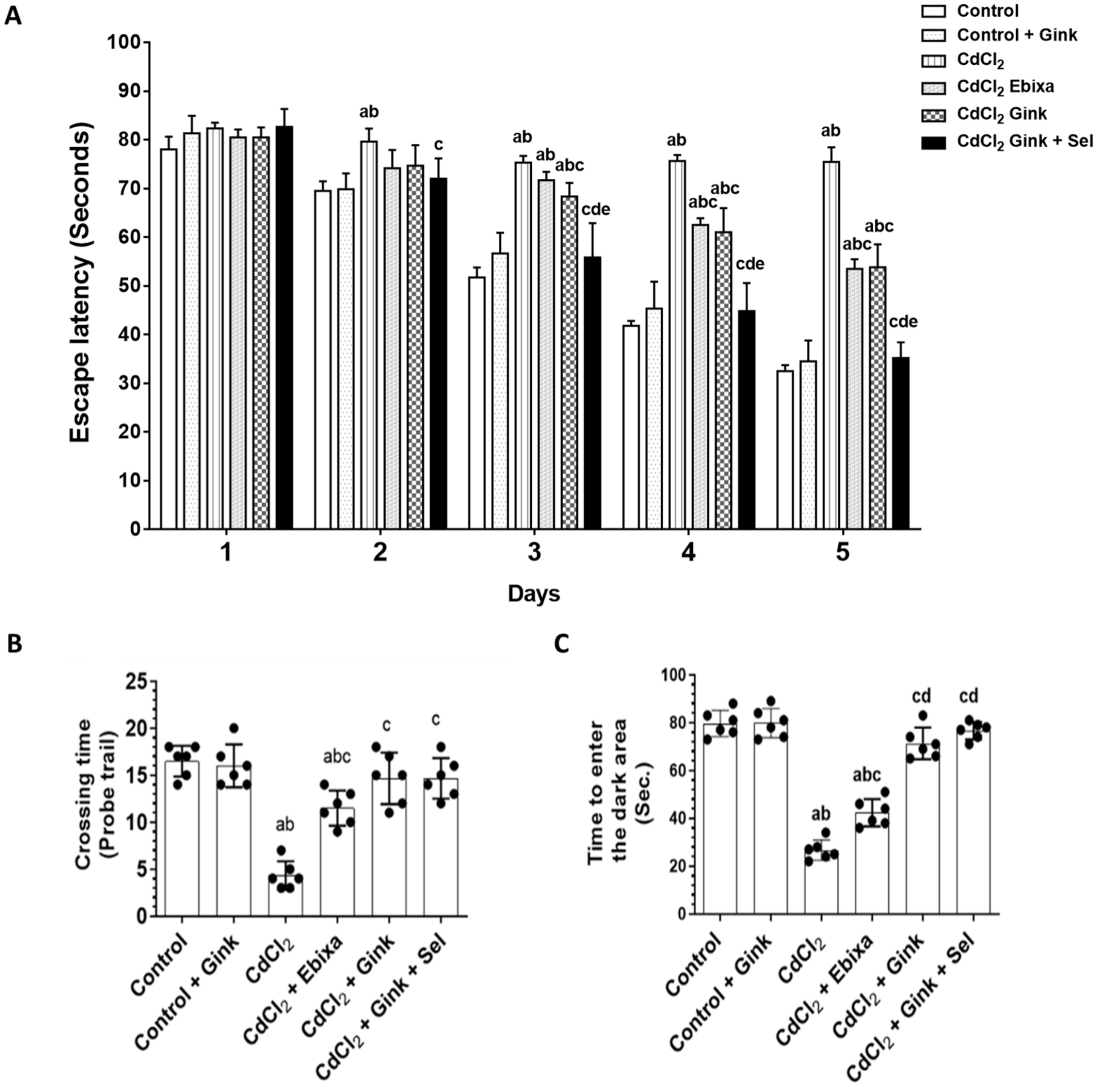
Effects of treatments on behavioral parameters. **(A)** The required time to find the hidden platform in the Morris water maze test. Data were expressed as mean ± SD for n = 6 rats/group. **(B)** The number of rats crossed over the removed hidden platform during the probe trial of the Morris water maze test. **(C)** The time required to enter the dark area during the passive avoidance learning test. Data were expressed as mean ± SD for *n* = 6 rats/group. a: Significantly different as compared to control group. b: Significantly different as compared to control + Ebixa group. c: Significantly different as compared to CdCl2 group. d: Significantly different as compared CdCl2 + Ebixa group. e: Significantly different as compared CdCl2 + *Ginkgo biloba* group. Gink, *Ginkgo biloba*; Sel, selenium.

In the Morris water maze test, significant differences were observed in the time required to find the hidden platform. These differences were notable when we compare the control, CdCl2 + *Ginkgo biloba* or CdCl2 + *Ginkgo biloba* + selenium groups data to that of CdCl2-treated rats. In addition, CdCl2 + Ebixa-treated rats also exhibited significant differences compared to the CdCl2 group. These significant changes were observed clearly starting from day 3 to day 5. Therefore, CdCl2 exposure had notable impacts on spatial memory and learning abilities, as depicted in Figure 1A.

### 3.2 The number of times the rats crossed

During the probe trial of the Morris water maze test, there were significant variations in the number of times rats crossed over the removed hidden platform. A decrease was apparent in the CdCl2-treated group as compared to control, or *Ginkgo biloba* groups, while CdCl2 + Ebixa-treated rats displayed reversing effect. In comparison to CdCl2 group, CdCl2 + *Ginkgo biloba*-treated rats or CdCl2 + *Ginkgo biloba* + selenium groups showed an increase in the number of crossing times (Figure 1B).

### 3.3 Time to enter the dark area

The time required for rats to enter the dark area in the passive avoidance learning test showed significant differences among various groups. CdCl2-treated rats had reduced spent time in the dark area compared to control and *Ginkgo biloba* groups, however, Ebixa treatments reversed this effect. Moreover, *Ginkgo biloba* alone or in combination with selenium normalized CdCl2-decreased spent time in the dark area (Figure 1C).

### 3.4 Acetylcholine (Ach) levels in the hippocampus

**Figure 2:**
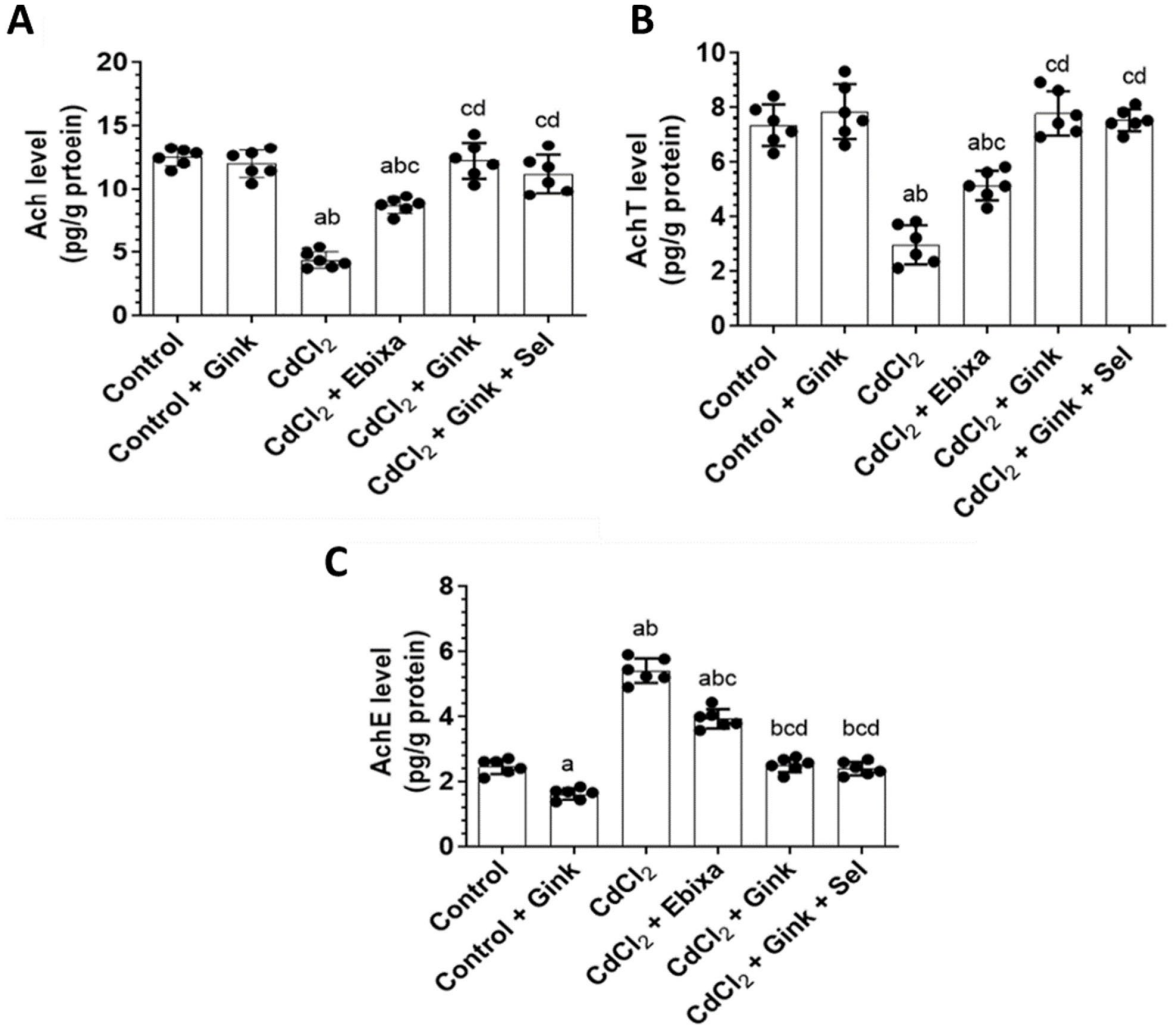
Effects of treatments on the hippocampus level of Ach, and hippocampal activity of AchT and AChE. **(A)** Levels of acetylcholine (Ach) in the hippocampus homogenates of all groups of rats. **(B)** Levels of acetylcholine transferase (AchT) in the hippocampus homogenates of all groups of rats. **(C)** Levels of acetylcholine esterase (AchE) in the hippocampus homogenates of all groups of rats. Data were expressed as mean ± SD for *n* = 6 rats/group. a: Significantly different as compared to control group. b: Significantly different as compared to control + Ebixa group. c: Significantly different as compared to CdCl2 group. d: Significantly different as compared CdCl2 + Ebixa group. Gink, *Ginkgo biloba*; Sel, selenium.

Levels of Ach in the hippocampus homogenates exhibited significant differences among all groups of rats. CdCl2-treated rats had decreased Ach concentration in the hippocampus as compared to the other studied groups. However, Ebixa treatments increased the hippocampal Ach levels in rats exposed to CdCl2, however, it was still lower than control or Ebixa alone groups. Similar effects were also observed with *Ginkgo biloba* alone or in combination with selenium. Interestingly, *Ginkgo biloba* or CdCl2 + *Ginkgo biloba* + selenium groups had higher Ach levels in the hippocampus as compared to CdCl2 + Ebixa group (Figure 2A).

### 3.5 Choline acetyltransferase (AchT) in the hippocampus

Activity of AchT in the hippocampus showed significant differences among all groups of rats. CdCl2-treated rats had reduced AchT activity in the hippocampus as compared to the other studied groups. However, Ebixa treatments elevated the AchtT activity in the hippocampus of rats exposed to CdCl2, however, AchtT activity was still lower than control or Ebixa alone groups. *Ginkgo biloba* alone or in combination with selenium treatments also reversed the effects of CdCl2 on the AchT activity in the hippocampus. Interestingly, *Ginkgo biloba* or CdCl2 + *Ginkgo biloba* + selenium groups had elevated brain AchT activity as compared to CdCl2 + Ebixa group (Figure 2B).

### 3.6 Acetylcholinesterase (AChE) in the hippocampus

AChE hippocampal activity revealed significant changes among all studied groups. CdCl2-treated rats increased AChE hippocampal activity comparing to the other groups od rats. However, Ebixa treatments reduced the brain AChE activity in the hippocampus of rats exposed to CdCl2, however, the activity was still higher than control or Ebixa alone groups. *Ginkgo biloba* alone or in combination with selenium treatments could normalize the elevation of AChE hippocampal activity induced by CdCl2 in the brain. Interestingly, *Ginkgo biloba* or CdCl2 + *Ginkgo biloba* + selenium groups had decreased brain AchT activity in the hippocampus as compared to CdCl2 + Ebixa group (Figure 2C).

### 3.7 Histopathology

#### 3.7.1 Cerebral cortex’s section after Ebixa treatment

**Figure 3:**
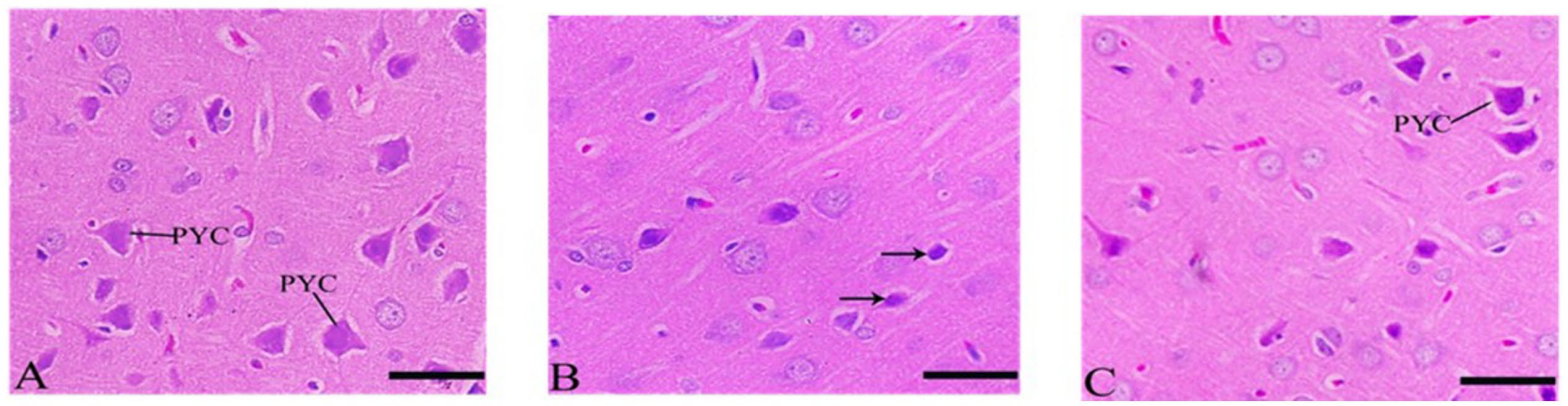
Histopathological sections of the cerebral cortex (the frontal lobe). **(A)** Control group, **(B)** CdCl_2_ group, **(C)** CdCl_2_ + Ebixa group (H & E stain). Scale bar = 50 μm. PYC, pyramidal cells.

Cerebral cortex sections showed inflammation in CdCl2 group compared to control group. Sections of the cerebral cortex showed a significant improvement in neuron in Ebixa + CdCl2 group as compared to CdCl2 only group (Figure 3).

#### 3.7.2 Cerebellum’s sections after Ebixa treatment

**Figure 4:**
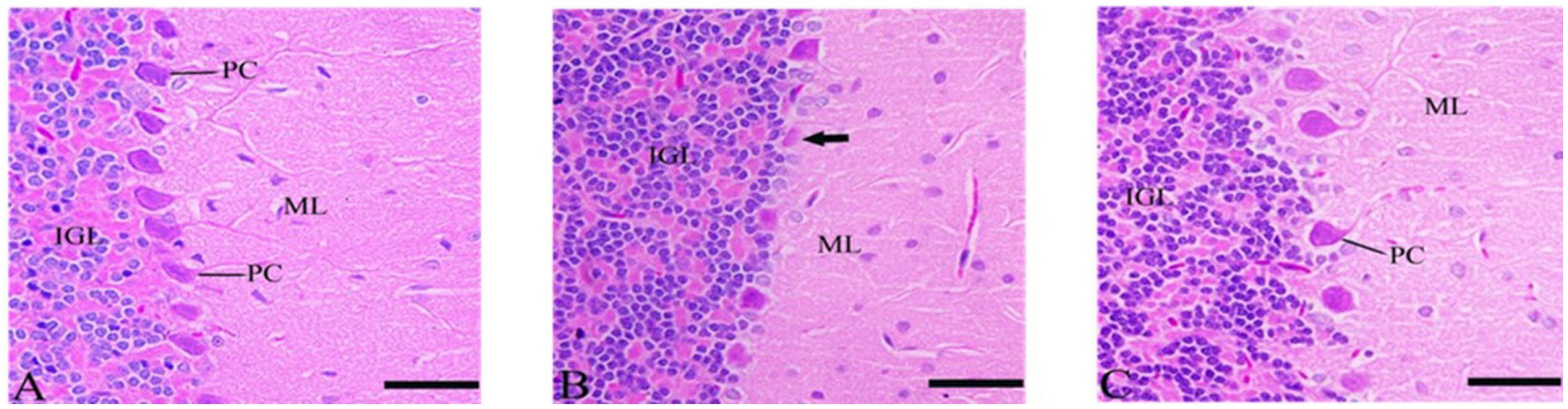
Histopathological sections of the cerebellum. **(A)** Control group, **(B)** CdCl_2_ group, **(C)** CdCl_2_ + Ebixa group (H & E stain). Scale bar = 50 μm. ML, molecular layer; IGL, inner granular layer; PC, Purkinje cell.

Purkinje cells appeared degenerated or abnormal in the CdCl2 group but showed significant neuronal protection in the CdCl2 + Ebixa group (Figure 4). An improvement in Purkinje neurons was also evident in the group after treatment by Ebixa in animals exposed to CdCl2, as shown in Figure 4.

#### 3.7.3 Sections of histological structures in medulla neuron after Ebixa treatment

**Figure 5:**
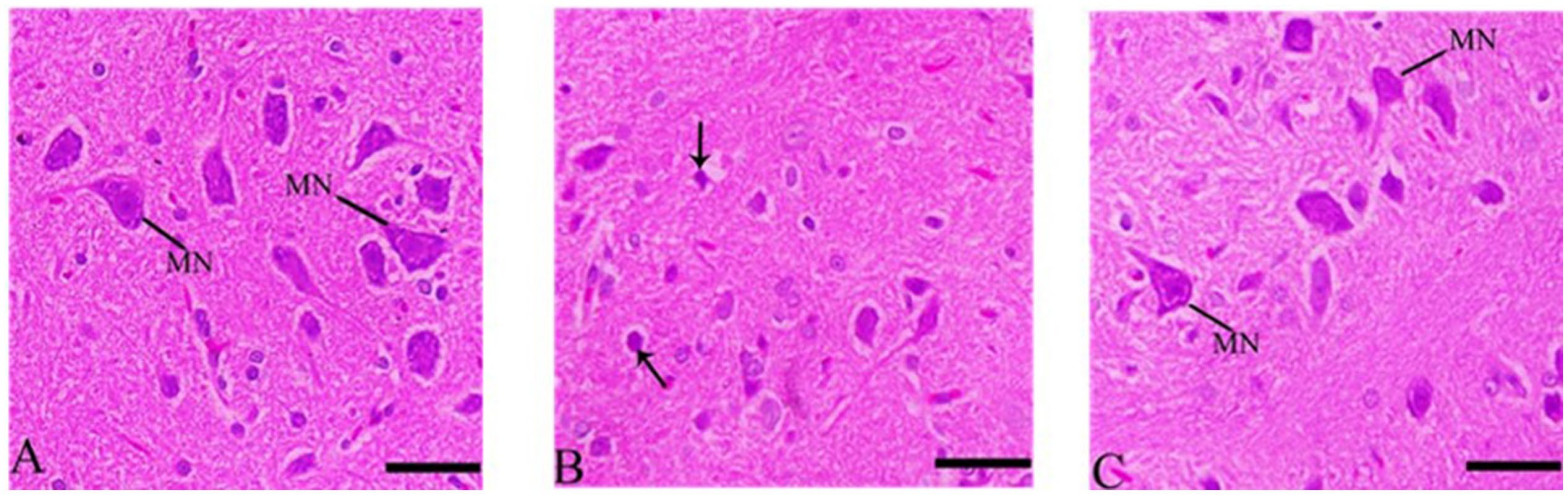
Histopathological sections of the medulla neurons (the lowest area of the brainstem). **(A)** Control group, **(B)** CdCl_2_ group, **(C)** CdCl_2_ + Ebixa group (H & E stain). Scale bar = 50 μm. MN, medullary neurons.

Most of the medulla neurons appeared small and pyknotic in the CdCl2 group. Ebixa treatment showed improvement in medulla neuronal structures, as depicted in Figure 5.

#### 3.7.4 Cerebral cortex’s sections after *Ginkgo biloba* and selenium treatments

**Figure 6:**
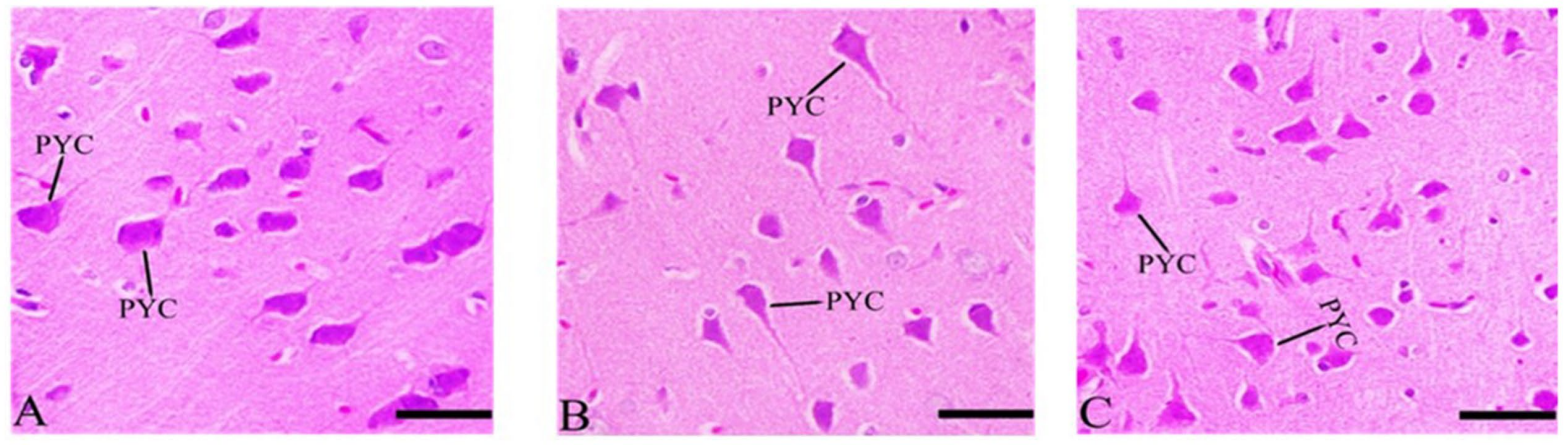
Histopathological sections of the cerebral cortex (the frontal lobe). **(A)** *Ginkgo biloba* group, **(B)** CdCl_2_ + *Ginkgo biloba* group, **(C)** CdCl_2_ + *Ginkgo biloba* + selenium group (H & E stain). Scale bar = 50 μm. PYC, pyramidal cells.

The rate of pyknosis in the cerebral cortex was reduced in the *Ginkgo biloba* group. *Ginkgo biloba* alone or in combination with selenium showed improvement in pyramidal cells when compared to the *Ginkgo biloba* group, resulting in significant neuronal improvement, as shown in Figure 6.

#### 3.7.5 Cerebellum’s sections after *Ginkgo biloba* and selenium treatments

**Figure 7:**
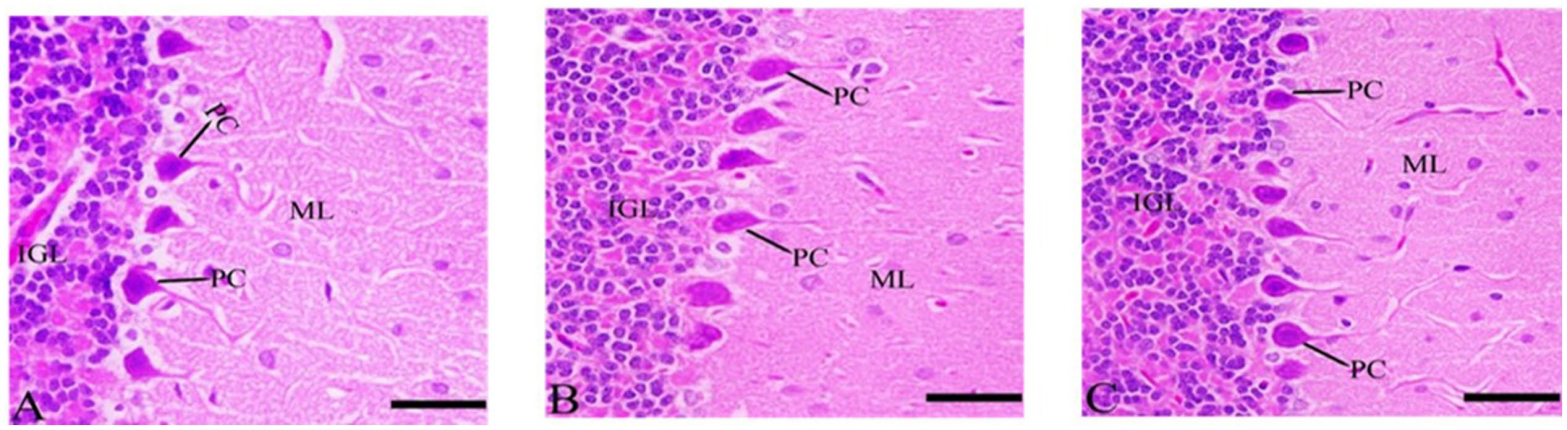
Histopathological sections of the cerebellum. **(A)** *Ginkgo biloba* group, **(B)** CdCl_2_ + *Ginkgo biloba* group, **(C)** CdCl_2_ + *Ginkgo biloba* + selenium group (H & E stain). Scale bar = 50 μm. ML, molecular layer; IGL, inner granular layer; PC, Purkinje cell.

In the CdCl2 + *Ginkgo biloba* group, protected neurons were observed, and no apoptosis was occurred. More protection was evident in the CdCl2 + *Ginkgo biloba* + selenium group as depicted in Figure 7.

#### 3.7.6 Histological structures in medulla neuron after *Ginkgo biloba* and selenium treatments

**Figure 8:**
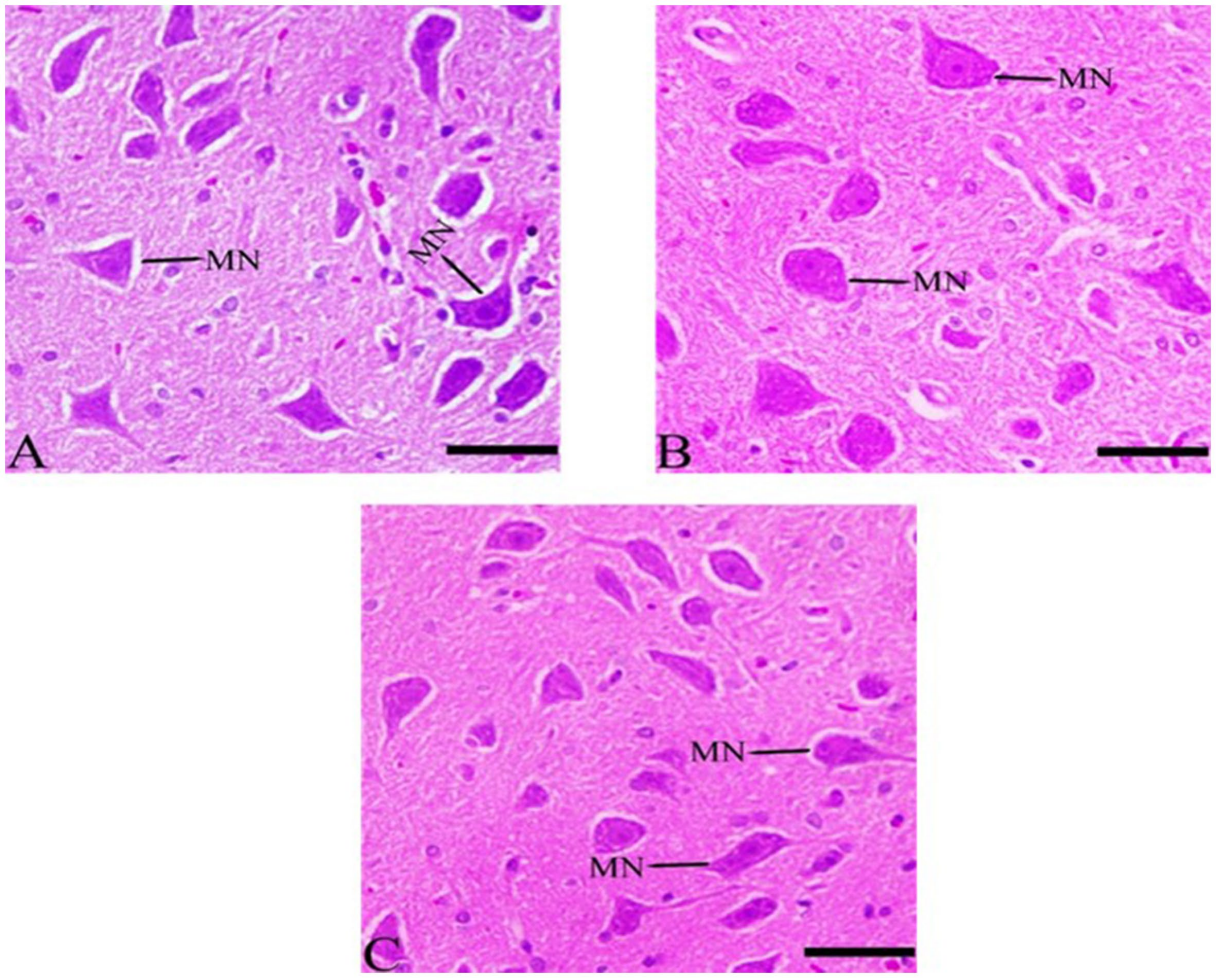
Histopathological sections of the medulla neurons (the lowest area of the brainstem). **(A)** *Ginkgo biloba* group, **(B)** CdCl_2_ + *Ginkgo biloba* group, **(C)** CdCl_2_ + *Ginkgo biloba* + selenium group (H & E stain). Scale bar = 50 μm. MN, medullary neurons.

The results indicate that the rate of pyknosis was reduced in CdCl2 + *Ginkgo biloba* group, indicating significant neuronal improvement. In addition, similar effects were observed in CdCl2 + *Ginkgo biloba* + selenium group, which show improvement in the pyramidal cells (Figure 8).

#### 3.7.7 Apoptotic (TUNEL assay) in the cerebral cortex after Ebixa treatment

**Figure 9:**
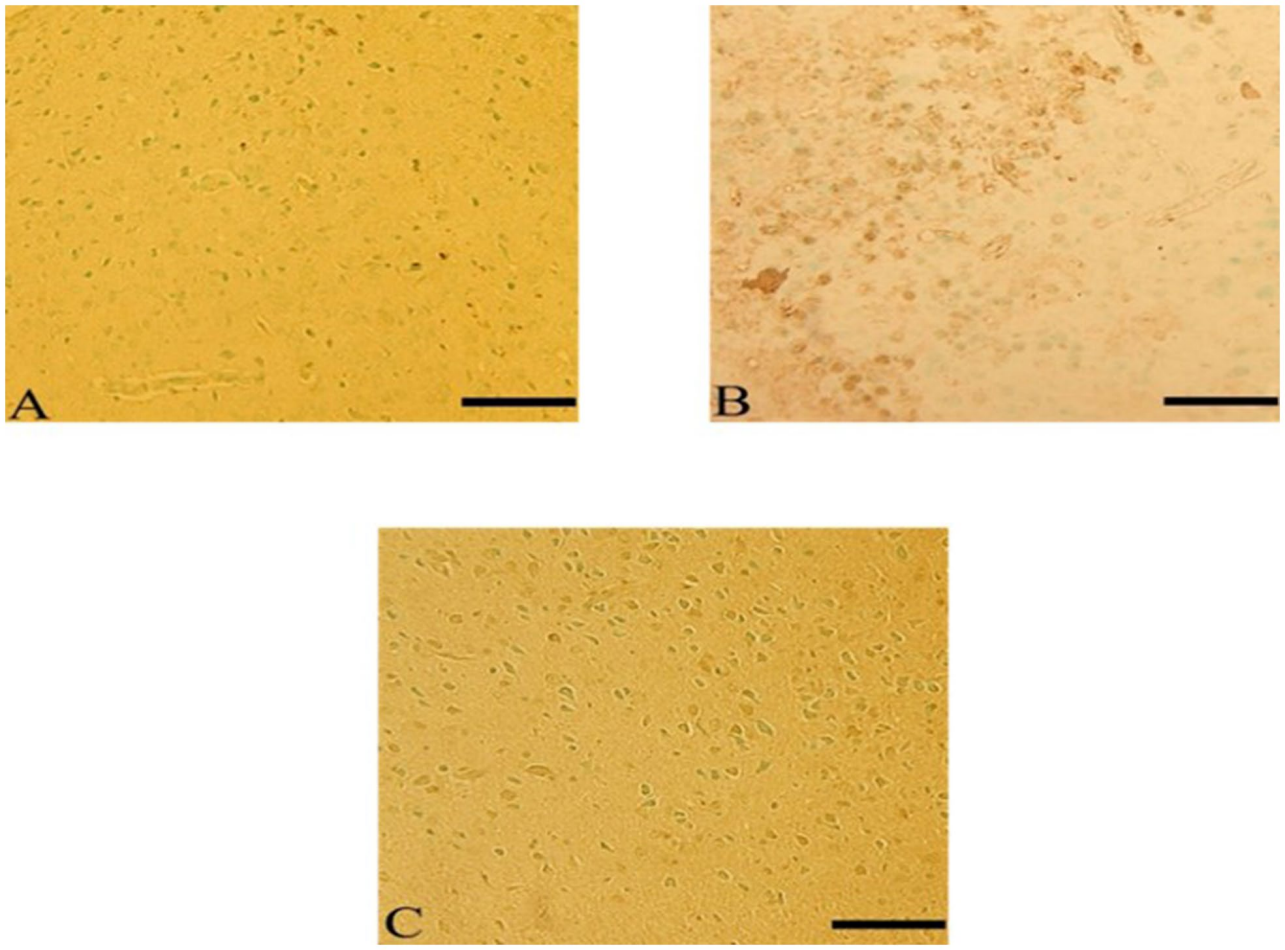
Ebixa reduced CdCl_2_-induced apoptotic changes in rats’ cerebral cortex. **(A)** Control group, **(B)** CdCl_2_ group, **(C)** CdCl_2_ + Ebixa group. TUNEL -positive cells appeared brown. Scale bar = 50 μm.

In the CdCl2 group, apoptotic cells in the cerebral cortex were significantly observed as compared to the control group. Ebixa treatment induced observable reductions in the apoptosis induced by CdCl2 treatment (Figure 9).

#### 3.7.8 Apoptotic (TUNEL assay) after *Ginkgo biloba* and selenium treatments

**Figure 10:**
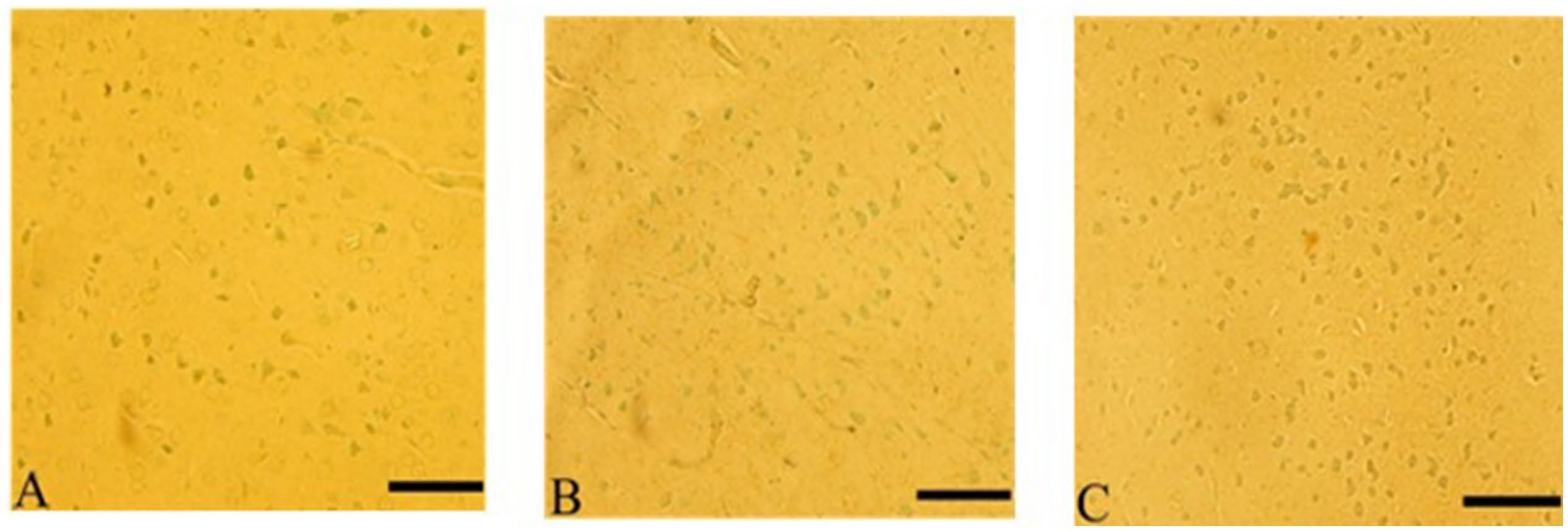
*Ginkgo biloba* and selenium reduced CdCl_2_-induced apoptotic changes in rats’ cerebral cortex. **(A)** *Ginkgo biloba* group, **(B)** CdCl2 + *Ginkgo biloba* group, **(C)** CdCl2 + *Ginkgo biloba* + selenium group. Scale bar = 50 μm.

*Ginkgo biloba* treatment protected neurons in the cerebral cortex from apoptosis-induced by CdCl2 exposure, and similar effects were observed in the CdCl2 + *Ginkgo biloba* + selenium group. These groups showed resistance to apoptosis compared to the CdCL2 group, as shown in Figure 10.

## 4 Discussion

The results of this study indicate that the administration of CdCl2 in rats had significant effects on various aspects of behavior, brain enzymes, and histological structures, mirroring the characteristics of the AD. These parameters include memory functioning, cholinergic system, neuroinflammation and neuronal death. Importantly, treatments with *Ginkgo biloba* alone or in combination with selenium exhibited varying degrees of efficacy in mitigating these effects. Ebixa also showed positive effects against these behavioral and molecular impairments-induced by CdCl2 exposure. *Ginkgo biloba* alone or in combination with selenium reversed CdCl2-induced memory impairments, an effect associated with normalizing Ach level as well as AchT and AChE activities in the brain. Inflammation and apoptosis were also attenuated in the brains of animals exposed to CdCl2.

In the Morris water maze test, CdCl2-treated animals displayed impaired spatial memory and learning abilities, characterized by an increased time required to find the hidden platform (Halder et al., 2016; El-Kott et al., 2020b). This aligns with previous studies that have shown cadmium-induced cognitive deficits (Deng et al., 2023). Moreover, treatment with Ebixa (memantine) demonstrated a significant improvement in cognitive abilities (Zhou et al., 2019), suggesting its potential as a therapeutic option for ameliorating CdCl2-induced cognitive impairments. The results of the probe trial in the Morris water maze test revealed that CdCl2-treated rats had a reduced number of crossings over the removed hidden platform, indicating impaired memory recall. In contrast, CdCl2 + Ebixa-treated rats exhibited resistance to these memory deficits, moreover, CdCl2 + *Ginkgo biloba* + Selenium-treated rats showed a similar trend. These findings are consistent with studies that have highlighted the memory-enhancing effects of *Ginkgo biloba* (Ge et al., 2021) and selenium (Liu et al., 2025) indicating their potential effects in attenuating behavioral impairments associated with AD. The passive avoidance learning test demonstrated significant differences in the time required to enter the dark area. CdCl2-exposed rats exhibited a shorter latency period compared to the control group, indicating impaired avoidance memory, which is in line with the cognitive deficits seen in AD animal models (El-Kott et al., 2020a). Notably, CdCl2 + *Ginkgo biloba* + Selenium treatment resulted in increased avoidance memory compared to the CdCl2 + *Ginkgo biloba* treated group, highlighting the potential neuroprotective effects of *Ginkgo biloba* and selenium.

Altered level of Ach in the brain areas is a critical characteristic of cognitive disorders associated with AD (Ismail et al., 2023). In this study, CdCl2 exposure led to a significant decrease in Ach levels in the hippocampus, while Ebixa treatment mitigated this effect. Notably, *Ginkgo biloba* and selenium co-administration also led to lower Ach levels, indicating their potential in modulating cholinergic neurotransmission associated with AD-like characteristics in rats. AchT is an essential enzyme for Ach synthesis and maintenance of cholinergic function (Oda, 1999; Bagwe and Sathaye, 2022). The CdCl2 + *Ginkgo biloba* and CdCl2 + *Ginkgo biloba* + selenium groups exhibited an increase in AchT levels in the hippocampus compared to the CdCl2, suggesting that *Ginkgo biloba* and selenium may have a stimulating effect on cholinergic neurons. Therefore, this finding explains the previous results showing increased brain Ach levels after treatment with *Ginkgo biloba* alone or with combinations with selenium. However, these findings require further investigation to elucidate the underlying mechanisms and responsible signaling pathways. AChE is a key enzyme responsible for Ach degradation and is often elevated in neurodegenerative diseases (McGleenon et al., 1999; Vecchio et al., 2021). In this study, AChE levels in the hippocampus were significantly decreased in the control + *Ginkgo biloba* and CdCl2 + *Ginkgo biloba* + selenium group compared to CdCl2 groups, indicating a potential role for *Ginkgo biloba* in modulating cholinergic activity. Conversely, CdCl2 treatment led to an increase in AChE, while CdCl2 + Ebixa resulted in a notable decrease. These findings emphasize the efficacy of Ebixa in alleviating AChE alterations, in addition to its blocking effect on NMDA receptor. It is important to note that *Ginkgo biloba* extract terpene trilactones found in ginkgo, such as ginkgolides A, B, C, and bilobalide, are responsible, among others, for its anti-epileptic activity on neurons in the hippocampus of the brain, which enhances memory and learning capacity and reduces neuronal damage (Biernacka et al., 2023). Moreover, *Ginkgo biloba* extract contains flavonoids that might have potential anti-oxidant, anti-inflammatory and neuroprotective effects (Nowak et al., 2021).

The histological analysis of different brain regions further supports the cognitive and neuroprotective effects of the *Ginkgo biloba*, selenium and Ebixa. CdCl2 + Ebixa group had significant neuronal improvement in the cerebral cortex. This improvement highlights the potential effects of Ebixa in preserving cortical neurons, possibly by regulating cholinergic signaling pathways observed in our current study. Purkinje cells in the cerebellum displayed signs of degeneration in the CdCl2 group, but Ebixa treatment offered significant neuronal protection suggesting additional promising effect for Ebixa. The cerebellum plays a crucial role in motor coordination and cognitive functions, and the preservation of Purkinje cells is essential for these processes (Zhang et al., 2023; Zobeiri and Cullen, 2024).

The medulla neurons showed signs of pyknosis and shrinkage in the group exposed to CdCl2. However, Ebixa treatment resulted in the improvement of medulla neurons, emphasizing its potential for preserving vital brainstem structures. *Ginkgo biloba* treatment in the Alzheimer led to a reduction in pyknosis and improved the condition of pyramidal cells in the cerebral cortex in animals exposed to CdCl2. This is in line with previous research suggesting the neuroprotective effects of *Ginkgo biloba* (Ge et al., 2021). Similar to the cerebral cortex, *Ginkgo biloba* treatment resulted in a reduction in pyknosis and the preservation of Purkinje cells in rats exposed to CdCl2. This neuroprotective effect indicates the potential effects of *Ginkgo biloba* in maintaining the histological characteristics cerebellar areas. The apoptotic analysis showed that Ebixa treatment prevented apoptosis induced by CdCl2, moreover, *Ginkgo biloba* alone or in combination with selenium produced similar effects in animals exposed to CdCl2. These results suggest that both Ebixa, *Ginkgo biloba* and the combination of *Ginkgo biloba* and selenium have anti-apoptotic effects in animals developed AD-like characteristics suggesting that testing these compounds against neuronal death and AD progression in animal models is crucial.

In conclusion, this study highlights the potential therapeutic benefits of Ebixa and *Ginkgo biloba* alone or in combination with selenium in mitigating cognitive deficits and preserving brain structures in a CdCl2-induced AD-like characteristics in rats. These findings provide valuable insights into the potential effects of these compounds on the behavioral and molecular changes associated with AD in animals. Further research is required to highlight the signaling pathways in the brain involved in mediating these positive effects. Importantly, memantine, selenium, and *Ginkgo biloba* can interact in complicated ways, affecting antioxidant defenses, enzyme activity, and neurological function. More investigation is required to completely comprehend these interactions and their consequences for therapeutic and harmful consequences, especially in the setting of neurological disorders. There is less research on the immediate protein-level interactions between Selenium and memantine. Both, however, are recognized to have an impact on cellular pathways associated with neurotransmitters, oxidative stress, and inflammation, all of which are important in neurodegenerative illnesses. As an antioxidant, selenium may help reduce some of the oxidative stress that can contribute to Alzheimer’s disease and may also boost the overall neuroprotective benefits of memantine or *Ginkgo biloba*.

## Data Availability

The original contributions presented in the study are included in the article/supplementary material, further inquiries can be directed to the corresponding author.
